# Does Autologous Transfusion Decrease Allogeneic Transfusion in Liposuction Surgery of Lymphedema Patients?

**DOI:** 10.3389/fmed.2022.778230

**Published:** 2022-04-05

**Authors:** Linfeng Chen, Kun Chang, Yan Chen, Zhenhua Xu, Wenbin Shen

**Affiliations:** ^1^Department of Blood Transfusion, Beijing Shijitan Hospital, Capital Medical University, Beijing, China; ^2^Department of Lymph Surgery, Beijing Shijitan Hospital, Capital Medical University, Beijing, China; ^3^HealSci Technology Co. Ltd., Beijing, China

**Keywords:** autologous transfusion, allogeneic transfusion, lymphedema, liposuction surgery, transfusion patterns

## Abstract

**Background and Objective:**

Liposuction is an effective treatment for fat disposition in lymphedema. Blood transfusion has been seldom investigated in lymphedema liposuction surgery. The purpose of the study was to analyze clinical factors associated with blood transfusion in liposuction surgery of lymphedema patients and compare the autologous and allogeneic transfusion patterns.

**Methods:**

A total of 1,187 cases of liposuction due to lymphedema were recruited. Demographic, laboratory tests and operation information were collected. Patients were divided into a transfusion and a non-transfusion group. Different transfusion patterns were compared and analyzed.

**Results:**

Between the two groups, there is a significant difference in postoperative hemoglobin levels, and as well as gender, age, surgery duration, body weight change, intraoperative transfusion volume and blood loss, hospital length of stay, and surgical site distribution. There is a significant difference in the comparison of hospital stay length, autologous transfusion volume, combined allogeneic volume, operative blood loss, intraoperative transfusion volume, and change in hemoglobin levels between predonation and acute normovolemic hemodilution (ANH) transfusion. In comparison with the allogeneic transfusion-only patients, the mean allogeneic transfusion volume in either ANH group, predonated transfusion group, or mixed group is statistically lower. Allogeneic transfusion volume in the predonated-only group is significantly lower than that of either the ANH-only group or the mixing ANH with predonation group. Ordinary least squares regression analysis suggests that autologous transfusion in the ANH-only mode is statistically associated with allogeneic transfusion.

**Conclusions:**

This study described the blood transfusion in lymphedema liposuction surgery and compared autologous and allogeneic transfusion patterns in these patients. Autologous transfusion can reduce the transfusion volume of allogeneic blood and might be a beneficial mode of transfusion in these patients.

## Introduction

Lymphedema is a chronic condition in which fluids and fibers accumulate in bodies due to primary or secondary lymphatic obstructions (1). The manifestation of lymphedema includes swelling, pain, and skin hardening. Although it is an incurable disease, multiple therapies can help to release the symptom and improve the quality of life. The treatment for lymphedema can be mainly divided into nonsurgical techniques (2), such as exercise and bandage, and surgical techniques (3), such as anastomosis. In many cases, the deposited adipose tissue might not be reduced merely through bandaging or anastomosing the lymphatic duct. Therefore, liposuction is often needed to complement the reconstruction process (4).

Liposuction is a technique that can remove excess fat tissues with suction-pump devices. In plastic surgery, it is generally used to reduce localized adipose tissue within the abdomen, breast, upper limbs, and lower limbs (5). Blood loss and fluid management have always been a major issue in the process of liposuction (6). To minimize blood loss, Illouz (7) in the 1970s first introduced the concept of “wet liposuction” with a small amount of diluted hyaluronidase and normal saline injected subcutaneously. The wet technique is still accompanied by significant volumes of blood loss. Later, in the 1980s, dermatologists modified the process by pumping fluid containing saline and epinephrine into the area preoperatively (8). This produces tumescent and vasoconstriction effects, thus further lowering the blood loss.

Although tumescent technique has made it safer for surgeons to practice liposuction, hemodynamic stability and blood loss are still the main considerations during the operation that can influence aspirated volumes (9). Under some circumstances, especially when the aspirated volume exceeds 1,500 ml, blood transfusion is required to correct hypoxic symptom or coagulation abnormalities in the patients. To the best of the authors' knowledge, there are very few articles that investigate blood transfusion in liposuction surgery of lymphedema patients to date.

Therefore, this study was designed to describe and detect significant clinical factors associated with blood transfusion in lymphedema liposuction surgery. Meanwhile, different modes of transfusion (i.e., autologous or allogeneic) were analyzed to explore the best transfusion modes for liposuction surgery in lymphedema patients.

## Methods

### Study Design, Participant, and Data Collection

A retrospective study design was adopted. Patients underwent liposuction in the Department of Lymphatic Surgery of Beijing Shijitan Hospital, Capital Medical University, from January 1, 2016 to August 31, 2019, were recruited as study population. Transfusion records and medical documents were retrospectively examined to extract the demographic characteristics (age, gender, and body weight), clinical data (diagnosis, admission date, and length of stay), laboratory test [pre- and postoperative prothrombin time (PT), activated partial thromboplastin time (APTT), fibrinogen (FIB), and hemoglobin (Hb)], and surgery-related information (intraoperative blood loss, autologous or allogeneic transfusion volume, transfusion component, operation duration, and surgical site). The collection was conducted by two independent investigators to ensure the exactness and correctness of data. The ethics of this study was discussed and approved by the local committee for the protection of patient's privacy at Beijing Shijitan Hospital.

### Transfusion Implementation Protocol

According to the regulation enacted by Beijing Shijitan Hospital, patients are not allowed to transfuse allogeneic red blood cell (RBC) until intraoperative blood loss exceeds 600 ml under a normal preoperative hemoglobin level and coagulation function. The autologous transfusion is not dictated by this regulation. Plasma transfusion is performed when the intraoperative RBC transfusion volume is >4 U. For the postoperation management, the indication for transfusing RBC is hemoglobin <80 g/L in combination with high content of RBC in drainage fluid or occurrence of hypoxia symptoms. The indication of transfusing plasma is as follows: (1) drastic bleeding (been transfused with RBC at 40–80 mg/kg within 24 h); (2) bleeding (infiltration of blood out of the wound, large volume of drainage fluid, PT extension >3 s, APTT extension >1.5 times or international normalized radio, INR >2); (3) disseminated intravascular coagulation (DIC); and (4) complicated with hepatic diseases. Predonated autologous transfusion was conducted in accordance with the guideline of the American Association of Blood Banks (AABB) whose indication is preoperative Hb > 110 g/L, hematocrit > 0.33, normal coagulation time, and normal cardiopulmonary, hepatic and renal function. A total of 200–400 ml of whole blood was used for predisposition. Concerning the intraoperative acute normovolemic hemodilution (ANH), two venous routes were created after anesthesia. One route is used for blood collection at 200 ml every 5 min. Collection indication is hematocrit >25%, albumin >30 g/L, and Hb around 100 g/L. Collection volume ranged from 200 to 1,600 ml. The other route is used to transfuse the plasma exchange fluid.

### Primary and Secondary Measurements

Our primary measurements were age, gender, surgical site, body weight change, surgery time, preoperative Hb level. These variables were used to assess their relationship with blood transfusion. The secondary measurement was transfusion volume and different transfusion modes. Correlation was evaluated between these variables.

### Statistical Analysis

Statistical analyses were performed through the use of Python 3.6 software. Normality distribution was confirmed by Kolmogorov–Smirnov test with “scipy” package (version 1.2.0). To describe nonnormally distributed continuous data, median and percentiles (25th and 75th) were presented, whereas mean and standard deviation (SD) were calculated to represent normally distributed data (package “tableone,” version 0.6.0). Nonparametric (Kruskal–Wallis test) and parametric methods (*t*-test) were adopted depending on distribution and equal variance. Box-Cox method was tentatively used to transform deviated data into normal distribution. For categorical data, chi-square test was used to detect the difference. Correlation was determined using Pearson's or Spearman's analysis depending on data type (continuous or categorical) and distribution (normal or nonnormal). Ordinary least squares (OLS) regression analysis was utilized to further explore the factors that can statistically influence allogeneic transfusion. Allogeneic transfusion volume was regarded as dependent variable whereas different modes of autologous transfusion volumes were independent variables. Intraoperative blood loss was adjusted with the three modes of autologous transfusion (predonated only, ANH only, and mixed). Statistical significance was set at two-tailed *p* < 0.05.

## Results

### Patient's Characteristics

From January 2016 to August 2019, there were 3,467 cases of lymphatic surgery, which include 856 cases of thoracic duct plasty, 671 cases of venous anastomosis, 614 cases of lymphangiography, 1,187 cases of liposuction, and 139 cases of other surgeries. The liposuction surgery had a higher rate of intraoperative blood transfusion (417/1187, 35.1%), which accounts for 96.4% of the entire perioperative blood transfusion volume of all surgeries. Other surgeries than liposuction had an intraoperative blood transfusion rate of 1–14%. Few patients had intraoperative plasma transfusion. According to the China's transfusion guideline, 1 U (150 ml) of RBC is defined as RBC products extracted from 200 ml whole blood. Based on whether to implement RBC transfusion or not, patients underwent liposuction were divided into transfusion and nontransfusion groups. The demographic and clinical characteristics of the two groups are shown in Table 1.

**Table 1:** Summary of patients' demographic and clinical information.

**Items**	**Nontransfusion group**	**Transfusion group**	***p-*value**	**Statistic**
Gender			<0.001	Chi-square
Female, *n*, (%)	726 (94.3)	352 (84.4)		
Male, *n*, (%)	44 (5.7)	65 (15.6)		
Age, years, [median (range)]	54 (3, 81)	53 (12, 82)	0.04	Kruskal–Wallis
Length of stay, days, [median (range)]	7 (1, 30)	7 (5, 47)	<0.001	Kruskal–Wallis
Surgical site			<0.001	Chi-square
Upper limb, *n*, (%)	301 (43.0)	21 (4.3)		
Lower limb, *n*, (%)	384 (54.8)	456 (93.6)		
Others, *n*, (%)	15 (2.2)	10 (2.1)		
Intraoperative transfusion volume, ml, [median (range)]	0 (0, 0)	400 (200, 2200)	<0.001	Kruskal–Wallis
Intraoperative blood loss, ml, [median (range)]	400 (0, 1500)	800 (0, 2000)	<0.001	Kruskal-Wallis
Body weight change, Kg, [median (range)]	5.5 (0.0, 38)	11.1 (0, 35.4)	<0.001	Kruskal-Wallis
Surgery duration, min, (mean ± SD)	152.4 ± 78.2	235.0 ± 96.8	<0.001	Two-Sample *t*-test
Preoperative Hb, g/L, *n*, (%)			0.789	Chi-square
<110	23 (3.1)	13 (3.6)		
≥110	715 (96.9)	345 (96.4)		
Postoperative Hb, g/L, *n*, (%)			<0.001	Chi-square
80–100	129 (17.8)	104 (29.0)		
<80	12 (1.7)	12 (3.3)		
≥100	584 (80.6)	243 (67.7)		

*A total number of 1,187 patients underwent liposuction surgery were recruited. Data are expressed as mean ± SD or median and percentile unless otherwise stated. Body weight change equals preoperative minus postoperative. Surgery duration was transformed by Box-Cox into normal distribution and then tested*.

There is no statistical significance in the comparison of preoperative Hb concentrations between transfusion and nontransfusion groups. However, there is significant difference in postoperative Hb levels, and also gender, age, surgery duration, body weight change, intraoperative transfusion volume, intraoperative blood loss, hospital length of stay, and surgical site distribution. Intraoperative blood transfusion is statistically associated with longer surgical time (*p* < 0.001), larger body weight change (*p* < 0.001), and larger intraoperative blood loss (*p* < 0.001). Patients who received transfusion had a higher proportion of lower limb surgery compared with nontransfusion (93.6 vs. 54.8%, *p* < 0.001). Although the comparison of age is statistically significant, this significance is very borderline (*p* = 0.04) and the medians are quite approximate (53 vs. 54). The gender ratio is remarkably deviated, with female predominant to male in either transfusion or nontransfusion groups. Despite a less predominant gender constitution, male patient was more likely to transfuse than female patient (odds ratio = 3.05, *p* < 0.001).

### Correlation Between Intraoperative Blood Transfusion and Clinical Characteristics

**Figure 1:**
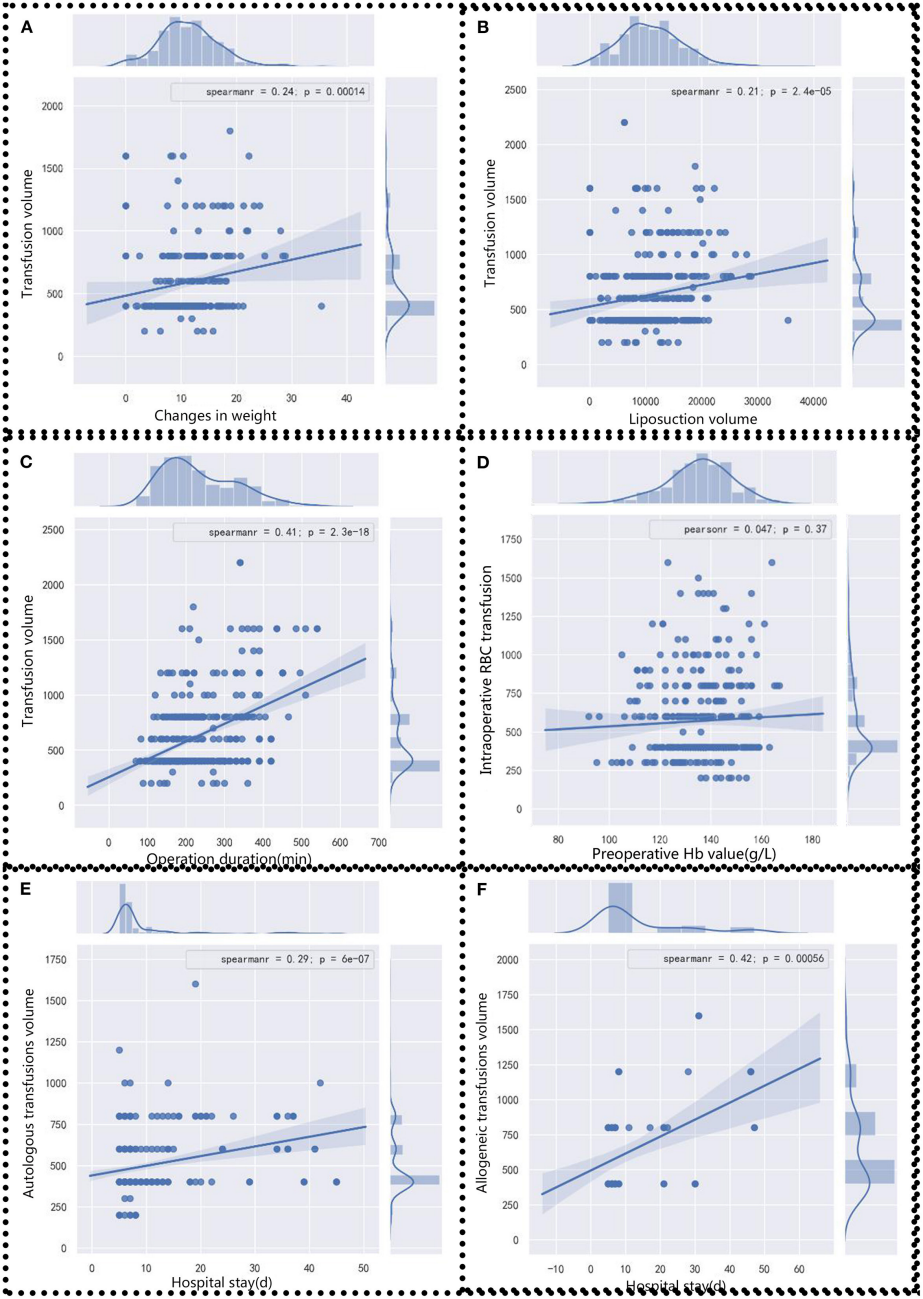
Correlation analysis between clinical factors and blood transfusion volume. Blood transfusion volumes were analyzed with **(A)** body weight change, **(B)** liposuction volume, **(C)** operation duration, and **(D)** preoperative Hb value. **(E)** Autologous and **(F)** allogeneic transfusions volumes were analyzed with the length of stay. Pearson's or Spearman's analysis was chosen according to data distribution. Statistical significance was assigned at *p* < 0.05.

To detect factors associated with intraoperative blood transfusion, several clinical factors that include body weight change, liposuction volume, surgery duration, preoperative Hb level, and length of hospital stay were analyzed through Pearson's or Spearman's correlation analysis. The body weight change value is positively correlated with blood transfusion volume (*r* = 0.24, *p* < 0.05) (Figure 1A). Similar correlations are also observed in intraoperative liposuction volume (*r* = 0.21, *p* < 0.05) (Figure 1B) and surgery duration (*r* = 0.41, *p* = 2.3E-18) (Figure 1C). There is statistical significance in preoperative Hb level (*r* = 0.047, *p* = 0.37) (Figure 1D). Although there appears to be a positive association between preoperative Hb and intraoperative RBC transfusion evidenced by the sloped line, the statistical association does not reach significant level, which suggests that there is no statistical significance in the comparison of association between the two variables. In other words, graph D does not implicate that the higher the preoperative Hb, the more the volume was. In terms of the length of stay with transfusion, the correlation is much stronger in allogeneic transfusion-only patients (*r* = 0.42) than in autologous transfusion-only patients (*r* = 0.29) (Figures 1E,F).

### Analysis of Autologous and Allogeneic Transfusion

The current investigation focuses on the liposuction surgeries since 2016, when we began to launch autologous blood transfusions, which include predonated autologous transfusion and intraoperative ANH. The intraoperative blood loss and intraoperative autologous or allogeneic transfusion volumes each year are summarized in Table 2.

**Table 2:** Intraoperative blood loss and transfusion volumes in liposuction surgery.

**Year**	**No. of surgery (*n*)**	**Intraoperative blood loss (ml)**	**Intraoperative blood transfusion (ml)**	**Autologous transfusion volume (ml)**	**Allogeneic transfusions volume (ml)**	**No. of predonated autologous transfusion only (*n*)**	**No. of intraoperative ANH only (*n*)**
2016	275	804 ± 412	463 ± 347	18,400 (61.13%)	11,700 (38.87%)	1	34
2017	301	368 ± 367	296 ± 260	34,700 (71.55%)	13,800 (28.45%)	3	90
2018	374	471 ± 256	178 ± 264	38,100 (77.91%)	10,800 (22.09%)	2	101
2019	337	489 ± 295	259 ± 319	47,600 (77.52%)	13,800 (22.48%)	56	45

*The volume of blood loss and transfusion that includes autologous and allogeneic transfusions was summarized. Data were detonated as mean ± SD or percentage*.

To compare the predonated autologous transfusion with intraoperative ANH transfusion, various indicators under the two scenarios were compared. As shown in Table 3, there was no statistical significance in gender (*p* = 0.108), age (*p* = 0.283), number of patients in combination with allogeneic transfusion (*p* = 0.942), blood coagulation index (*p* = 0.128 for PT, *p* = 0.514 for APTT, and *p* = 0.356 for FIB), and change in Hb level (in patients with combined transfusion with allogeneic) (*p* = 0.146). However, there was statistical significance in the comparison of length of hospital stay (*p* = 0.017), autologous transfusion volume (*p* < 0.001), combined allogeneic volume (*p* = 0.006), intraoperative blood loss (*p* < 0.001), intraoperative transfusion volume (*p* = 0.001), change in Hb level in transfusion patients (*p* < 0.001), and change in Hb level in patients with autologous transfusion only (*p* < 0.001).

**Table 3:** Comparison of autologous transfusion mode between acute normovolemic hemodilution-only patients and predonated autologous transfusion-only patients.

**Item**	**Acute normovolemic hemodilution-only patients (*n* = 270)**	**Predonated autologous transfusion-only patients (*n* = 56)**	***p-*value**	**Statistics**
Gender, *n* (%)			0.108	Chi-square
Male	45 (16.7%)	4 (7.1%)		
Female	225 (83.3%)	52 (92.9%)		
Age, *n* (%)			0.283	Chi-square
<40	60 (22.2%)	9 (16.1%)		
40–60	144 (53.3%)	28 (50.0%)		
>60	66 (24.4%)	19 (33.9%)		
Length of stay, days, [median (range)]	7.0 (5, 46)	7.0 (5, 21)	0.017	Kruskal–Wallis
Autologous transfusion volume, ml, [median (range)]	400.0 (200, 1600)	400.0 (200, 800)	<0.001	Kruskal–Wallis
Combination with allogeneic transfusion, n (%)			0.942	Chi-square
No	220 (81.5%)	46 (82.1%)		
Yes	50 (18.5%)	10 (17.9%)		
Combined allogeneic volume, ml, [median (range)]	400.0 (400, 1600)	400.0 (400.0, 400.0)	0.006	Kruskal–Wallis
Intraoperative blood loss, ml, [median (range)]	600.0 (0, 2000)	600.0 (10, 1000)	<0.001	Kruskal–Wallis
Intraoperative transfusion volume, ml, [median (range)]	400.0 (200, 2200)	400.0 (400, 800)	0.001	Kruskal–Wallis
**Change in coagulation indicators**				
Prothrombin time, PT, seconds, (mean ± SD)	1.5 ± 0.8	2.1 ± 0.6	0.128	Two-sample *t*-test
Activated partial thromboplastin time, APTT, seconds, (mean ± SD)	−3.6 ± 1.9	−3.2 ± 0.9	0.514	Two-sample *t*-test
Fibrinogen, FIB, seconds, (mean ± SD)	−0.4 ± 0.6	−0.7 ± 0.6	0.356	Two-sample *t*-test
Change in Hb levels in transfusion patients, g/L, (mean ± SD)	32.4 ± 14.0	23.8 ± 11.7	<0.001	Two-sample *t*-test
Change in Hb levels in autologous transfusion-only patients, g/L, (mean ± SD)	31.6 ± 12.7	23.4 ± 9.8	<0.001	Two-sample *t*-test
Change in Hb levels in patients of combined transfusion with allogeneic, g/L, (mean ± SD)	36.6 ± 19.3	25.8 ± 18.9	0.146	Two-sample *t*-test

*Change in Hb levels is calculated as preoperation minus postoperation, and change in coagulation indicators is calculated as postoperation minus preoperation. PT, APTT, FIB, and change in Hb level were transformed by Box-Cox into normal distribution and then compared through t-tests*.

These current results suggest that in patients with predonated autologous transfusion-only, the autologous transfusion volume, simultaneous allogeneic transfusion volume, and intraoperative transfusion volume are, respectively, lower than that of ANH-only patients. Although the median volume of intraoperative ANH transfusion is equal to that of predonated autologous transfusion, the range of intraoperative ANH transfusion volume is wider than that of predonated ones (200–1,600 vs. 200–800). In terms of the Hb levels, the decline of Hb is greater in the intraoperative ANH transfusion-only patients than in the predonated autologous transfusion-only patients. The range of length of hospital stay in the ANH-only patients is larger than that of predonated transfusion-only patients.

### Impact of Autologous Transfusion on Allogeneic Transfusion

**Figure 2:**
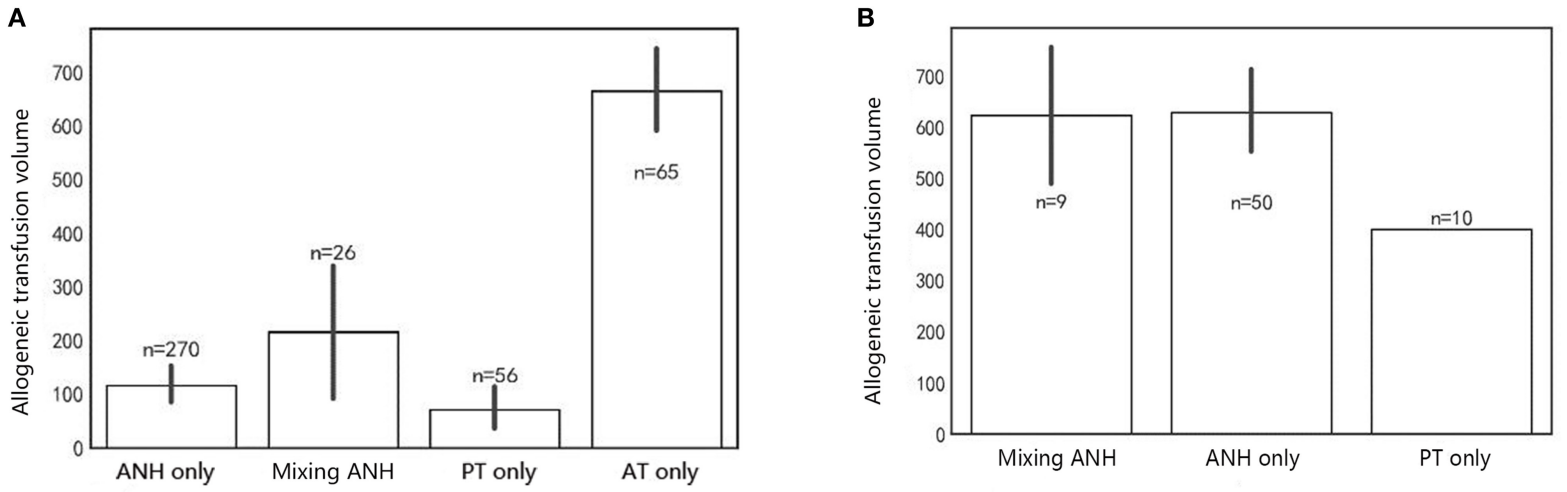
Allogeneic blood transfusion volume of different situations. **(A)** Total allogeneic blood consumption across different modes. **(B)** Combined allogeneic blood consumption across different modes. Bar represents mean ± SD. ANH, Acute normovolemic hemodilution; Mixing ANH, mixing acute normovolemic hemodilution with predonation; PT, predonated transfusion; AT, allogeneic transfusion.

The allogeneic blood transfusion volumes were analyzed across different transfusion modes to determine whether autologous transfusion could reduce the consumption of allogeneic transfusion. We observed that the allogeneic transfusion volume in the autologous transfused patients was less than that of the allogeneic transfusion-only patients. As suggested in Figure 2A, in comparison with the allogeneic transfusion-only patients, the mean allogeneic transfusion volume in either ANH group, predonated transfusion group, or mixed group is statistically lower (*p* < 0.001).

Since the autologous blood transfusion could reduce the allogeneic transfusion volume in liposuction surgery, we further compared the three modes of autologous transfusion by Kruskal–Wallis test to determine which mode is best (Figure 2A). There were 270, 56, and 26 cases in the intraoperative ANH-only group, predonated transfusion-only group, and combined transfusion of ANH and predonation groups, respectively. There is no statistical significance across the three groups concerning the allogeneic blood consumption (*p* = 0.103) (Figure 2A). The mean value of allogeneic transfusion volume in intraoperative ANH-only patients and predonated transfusion-only patients is <150 ml, which indicates small demands for allogeneic transfusion in autologous transfusion patients.

To explore its impact on allogeneic transfusion, allogeneic volumes in combined modes were listed separately (Figure 2B). Under the circumstance of mixed transfusion, the combined allogeneic transfusion volume was statistically significantly different among the three groups (Figure 2B) (*p* = 0.017). *Post hoc* tests suggested that the allogeneic transfusion volume in the predonated-only group was insignificantly lower than that of the ANH-only group (*p* = 0.06) but significantly lower than that of the mixing ANH with predonation group (*p* = 0.008). These results implicate that the demand for allogeneic volume is small. The comparison between the ANH-only group and the mixing ANH with predonation group was of no statistical significance (*p*=0.78). The mean allogeneic transfusion volume in the predonated-only group is the lowest.

Ordinary least squares regression results showed that ANH-only group had a significant correlation with allogeneic transfusion and the other two modes had no significance (Table 4). The coefficient in the equation is the highest in the ANH-only group, followed by mixed transfusion mode, and predonated-only mode.

**Table 4:** Ordinary least squares regression analysis between autologous and allogeneic transfusion.

**Item**	**Coefficient**	**Standard error**	***t***	***P*-value**	**95% CI**
Constant	283.425	61.589	4.602	0.000	159.780–407.070
Intraoperative blood loss	0.137	0.067	2.052	0.045	0.003–0.270
ANH only	147.393	36.251	4.066	0.000	74.616–220.170
Predonated only	9.835	49.900	0.197	0.845	−90.343–110.012
ANH + predonated	126.197	69.763	1.809	0.076	−13.858–266.253

*Intraoperative blood loss and three modes of autologous transfusion volumes (ANH only, predonated only, and ANH + predonated transfusion) were the independent variables, and allogeneic transfusion volume was dependent variables. ANH: acute normovolemic hemodilution*.

## Discussion

In this study, we found that transfusion group had a lower postoperative Hb levels, and blood transfusion was associated longer surgery time, larger body weight change, and larger operative blood loss. Intraoperative blood transfusion volume was correlated with body weight change, intraoperative liposuction, and surgery duration. Either autologous or allogeneic transfusion volume was positively associated with the length of stay. Between the ANH-only patients and predonated-only patients, there is significant difference in terms of the length of stay, autologous transfusion volume, mixed transfusion with allogeneic volume, intraoperative blood loss, intraoperative transfusion volume, and change in Hb levels. Predonated-only mode had the smallest demand for allogeneic transfusion compared with other two modes. OLS regression analysis suggests that autologous transfusion in the ANH-only mode is statistically associated with allogeneic transfusion.

To the best of our knowledge, this study has the largest sample size ever to describe blood transfusion in lymphedema liposuction surgery. Through PubMed search of English literature, we have found no article that investigates the blood transfusion in liposuction surgery for lymphedema patients. Thus, we venture the idea that our study is likely to be the first specific account of blood transfusion in lymphedema liposuctions. Previously, there seem to have been only two scattered investigations occasionally mentioning blood transfusion in lipoaspiration of lymphedema. Brorson et al. (10) described eight breast cancer-related lymphedema patients indicated for blood transfusion during liposuction. Wojnikow et al. (11) reported that a total of nine patients were subjected to lymphedema adipose suction receiving blood transfusion. However, their main focus was not on the blood transfusion itself, and they just utilized transfusion as an intermediate variable for the assessment of other treatments. The two articles simply brought up the occurrence of blood transfusion and have not specifically evaluated the effect of transfusion. Moreover, the small sample size <10 severely limits the ability tow further extend their conclusion. From the most optimistic point of view, our study that comprises the largest sample size of 1,187 patients will lend invaluable academic credence to clinicians or scholars worldwide.

The operation of liposuction usually causes a substantial loss of fluid or blood from circulation or the third space. In this context, it poses a major risk for surgeons to aspire more than 2,000 ml of adipofibrosis tissue in one-stage surgery (12). A lack of circulating fluid is very dangerous for the perfusion of cardiopulmonary organs. Fortunately, availability of blood transfusion would effectively minimize the complication associated with the liposuction surgery (13). In our study, we found a positive significant association between liposuction volume and blood transfusion volume. This is consistent with clinical practice since the necessity of transfusion increases as the liposuction volume progresses. There is a literature recommending autologous transfusion in large-volume liposuctions (14). Compared with allogeneic blood, autologous blood is obviously safer in that it is associated with less morbidity and is logically free from blood-transmitted diseases (15). In our hospital, we began to pay more attention to autologous transfusion since 2013, and the percentage of autologous volume out of all transfusion volumes in liposuction surgery increased from 53.85 to 77.52%. We have observed that the demand for allogeneic blood was lower in patients with autologous transfusion than that in allogeneic transfusion-only patients, which implies that autologous transfusion would decrease the consumption of allogeneic blood. Similar conclusions have been drawn in the field of orthopedics that autologous transfusion is a cost-effective method to reduce the need for and quantity of allogeneic transfusion in elective total knee arthroplasty (16). Apart from the substitutability to consumption, another benefit of autotransfusion is the close-to-zero risk of infecting transfusion-transmitted disease such as AIDS or malaria (17). The blood-borne inflectional diseases can be tested before the operation to verify the status of infection, which is important for both surgeon and patients.

There are two main types of autotransfusion: the ANH, which is conducted during operation without preoperative blood collection (18), and the predonated transfusion, which is prepared and collected preoperatively (19). In our study, we compared the two types of transfusion and found that predonation is associated with shorter hospital stay, lower blood loss, and smaller decrement of Hb change. This can be explained by that predonation usually begins 3–7 days before the surgery, which allows the body to have time to adjust to normal levels in advance and thus recover faster postoperatively (20). However, ANH does not have this advantage. Supporting evidence also emerges from our OLS analysis: by adjusting the intraoperative blood loss as a covariate, we discovered that the ANH, not predonation, has the correlation with allogeneic transfusion volume, which indicates that even taking into consideration of blood loss, ANH is still undesirably correlated with the use of allogeneic blood. In other words, in terms of reducing the allogeneic transfusion, ANH is not an ideal choice compared with predonation. Additionally, in the three modes of autotransfusion (predonation, ANH, and predonation + ANH) in our analysis, the predonation mode had the significantly lowest consumption for allogeneic blood than other two modes. This also further strengthens our idea in favor of predonated autotransfusion.

Another worthy point is that allogeneic blood transfusion is associated with immune modulations (21). Possible mechanisms of transfusion-associated immunomodulation are T-helper (Th) 2 activation and human leukocyte antigen (HLA) response (22). For instance, during allogeneic transfusion, Th2 cell is activated and the response of Th1 cell is thus decreased (23). Other mechanisms of immunomodulation might include the balance between T regulatory cell (Treg) and Th 17 cells: transfusion helped reverse the imbalance of Treg/Th17 ratios in hip fracture patients evidenced by the decrease and increased frequency of Tregs and Th17, respectively, after blood transfusion (24). In addition, in polytransfused sickle cell disease patients, high levels of CXCR5^+^PD1^+^CD4^+^ T lymphocyte (TL) may be a biomarker for the inhibited functions of T cells (25). Multiple rounds of exposure to antigens in allogeneic blood transfusion trigger low degree of inflammatory stimulation, and the mononuclear cells cannot be presented. In addition, the elevated level of IL-10 in serum of transfused patients also accounts for suppression of immune response (26). Transfusion can cause acute lung injury *via* the modulation of HLA antibodies, evidenced by the fact that exclusion of plasma volume products can reduce the incidence of acute lung injury by nearly two-thirds (27). In gastric cancer, allogeneic blood transfusion is a deleterious prognostic factor on cancer-related mortality and recurrence (28). Blood restriction management has been proposed to minimize the use of allogeneic blood in gastric cancer. However, in the field of liposuction surgery, there is no such proposition. We herein make a radical supposition that autologous blood transfusion should be advocated in comparison with allogeneic transfusion in liposuction surgery, especially considering the modulated immune system that might not be good for lymphedema patients.

There are several drawbacks associated with the study. One limitation is that the sample size might not be sufficient to draw a solid conclusion. However, it is unclear what sample size is statistically required, since the current investigation is only a pilot cross-sectional analysis. Furthermore, our study lacks fundamental basic mechanistic research. There is a need for molecular studies to illustrate the biological role of transfusion in liposuction of lymphedema patients. Last but not least, the long-term effect of transfusion after discharge from hospital remains undetermined. Further study to examine the long-term role of blood transfusion would be needed.

## Conclusions

This study described the blood transfusion in lymphedema liposuction surgery and compared autologous with allogeneic transfusion patterns in these patients. Autologous transfusion can reduce the transfusion volume of allogeneic blood.

## Data Availability Statement

The original contributions presented in the study are included in the article/supplementary material, further inquiries can be directed to the corresponding author.

## Ethics Statement

The studies involving human participants were reviewed and approved by Scientific Research Ethics Committee of Beijing Shijitan Hospital, Capital Medical University. The affiliation of the Ethics Committee: National Health Commission of the People's Republic of China. The patients/participants provided their written informed consent to participate in this study. Written informed consent was obtained from the individual(s) for the publication of any potentially identifiable images or data included in this article.

## Author Contributions

LC contributed to the design of the study, statistical analyses, patient recruitment, execution of the patients' measurements, and manuscript preparation. YC and KC contributed to patient recruitment, execution of the patients' measurements, and manuscript preparation. ZX contributed to the statistical analyses and manuscript preparation. WS contributed to the design of the study and manuscript preparation. All authors contributed to the article and approved the submitted version.

## Conflict of Interest

ZX was employed by HealSci Technology Co. Ltd. The remaining authors declare that the research was conducted in the absence of any commercial or financial relationships that could be construed as a potential conflict of interest.

## Publisher's Note

All claims expressed in this article are solely those of the authors and do not necessarily represent those of their affiliated organizations, or those of the publisher, the editors and the reviewers. Any product that may be evaluated in this article, or claim that may be made by its manufacturer, is not guaranteed or endorsed by the publisher.
